# Strong and robust polarization anisotropy of site- and size-controlled single InGaN/GaN quantum wires

**DOI:** 10.1038/s41598-020-71590-x

**Published:** 2020-09-21

**Authors:** Hwan-Seop Yeo, Kwanjae Lee, Young Chul Sim, Seoung-Hwan Park, Yong-Hoon Cho

**Affiliations:** 1 Department of Physics, Korea Advanced Institute of Science and Technology (KAIST), Daejeon, 34141 Republic of Korea; 2 Department of Electronics Engineering, Catholic University of Daegu, Kyeongsan, 38430 Republic of Korea

**Keywords:** Materials science, Nanoscience and technology, Physics

## Abstract

Optical polarization is an indispensable component in photonic applications, the orthogonality of which extends the degree of freedom of information, and strongly polarized and highly efficient small-size emitters are essential for compact polarization-based devices. We propose a group III-nitride quantum wire for a highly-efficient, strongly-polarized emitter, the polarization anisotropy of which stems solely from its one-dimensionality. We fabricated a site-selective and size-controlled single quantum wire using the geometrical shape of a three-dimensional structure under a self-limited growth mechanism. We present a strong and robust optical polarization anisotropy at room temperature emerging from a group III-nitride single quantum wire. Based on polarization-resolved spectroscopy and strain-included 6-band k·p calculations, the strong anisotropy is mainly attributed to the anisotropic strain distribution caused by the one-dimensionality, and its robustness to temperature is associated with an asymmetric quantum confinement effect.

## Introduction

Control of optical polarization is essential for photonic applications including optical image encryption^1^, three-dimensional (3D) imaging^2^, and polarization-based visible light communication^3^. Because these applications demand an orthogonality of the polarization as the degree of freedom of information, a strongly polarized high-efficiency emitter becomes a substantial building block for an improved performance.

Group III-nitride-based quantum wells (QWs) on the non-polar plane (*m*- or *a*-plane) of a Wurtzite crystal are attractive candidates, owing to their high brightness and strong polarization anisotropy^4–7^. Their polarization anisotropy stems from anisotropic biaxial strain caused by the reduced in-plane symmetry, strongly decoupling the valence band structures into each orthogonal polarization (x and y) with a finite energy difference. A larger energy difference provides a robust degree of linear polarization of the emissions at room temperature. However, achieving high-quality non-polar QWs remains difficult, and expensive non-native substrates are obstacles for practical device applications^8^. Moreover, a polarized light emission, starting from unpolarized emissions of the QWs on the polar-plane (*c*-plane), e.g., elliptical nanorods or nano-gratings through a top-down approach, has been attempted^9,10^. These QWs demonstrate a degree of polarization (~ 71%) through asymmetric nanostructures of a few hundred nanometers^10^. More asymmetry is necessary for achieving a higher polarization, which is difficult using a top-down approach because of the effort and costs for reducing the nanostructures to less than a few tens of nanometers.

Another option is the use of one-dimensional (1D) quantum wires (QWRs) with a diameter of a few nanometers through a bottom-up approach. QWRs exhibit a polarization anisotropy owing to their 1D properties, including an asymmetric quantum confinement^11,12^ and non-biaxial strain distribution^13^. The formation of GaN/AlN QWRs have been reported on the *m*-plane^14–16^. Using III-nitride QWRs formed by a bottom-up approach, the optical polarization of the emission of the GaN QWRs formed on the *m*-plane^17^, ultraviolet lasing with self-assembled GaN nanowires encapsulated by AlGaN^18^ and complex carrier dynamics with six-QWRs formed at the edge of self-assembled hexagonal GaN/AlN nanowires have been investigated^19,20^. The mutual manipulation of the color and the polarization of light was reported using the InGaN QWR network in a single GaN microrod with the degree of polarization of ~ 55% at room temperature^21^. The growth controllability and the improvement of the polarization anisotropy of the QWR are essential for the practical photonic applications based on conventional *c*-plane substrates.

Herein, we demonstrated a strong and robust polarization anisotropy of a group III-nitride QWRs at room temperature. We present a bottom-up fabrication method for site-selective and size-controlled single InGaN QWRs, based on the geometrical shape of the 3D structure under a self-limited growth mechanism. We exploit the crystallographic in-plane symmetry of the *c*-plane of a Wurtzite crystal to obtain a relationship between optical polarization anisotropy and a 1D geometry. We characterized a size-dependent polarization anisotropy through a polarization-resolved micro-photoluminescence (μ-PL) spectroscopy and compared it with the 6-band k·p calculation including the effect of non-biaxial strain.

### Formation of single InGaN/GaN quantum wire grown on 3D structure under self-limited growth regime

**Figure 1:**
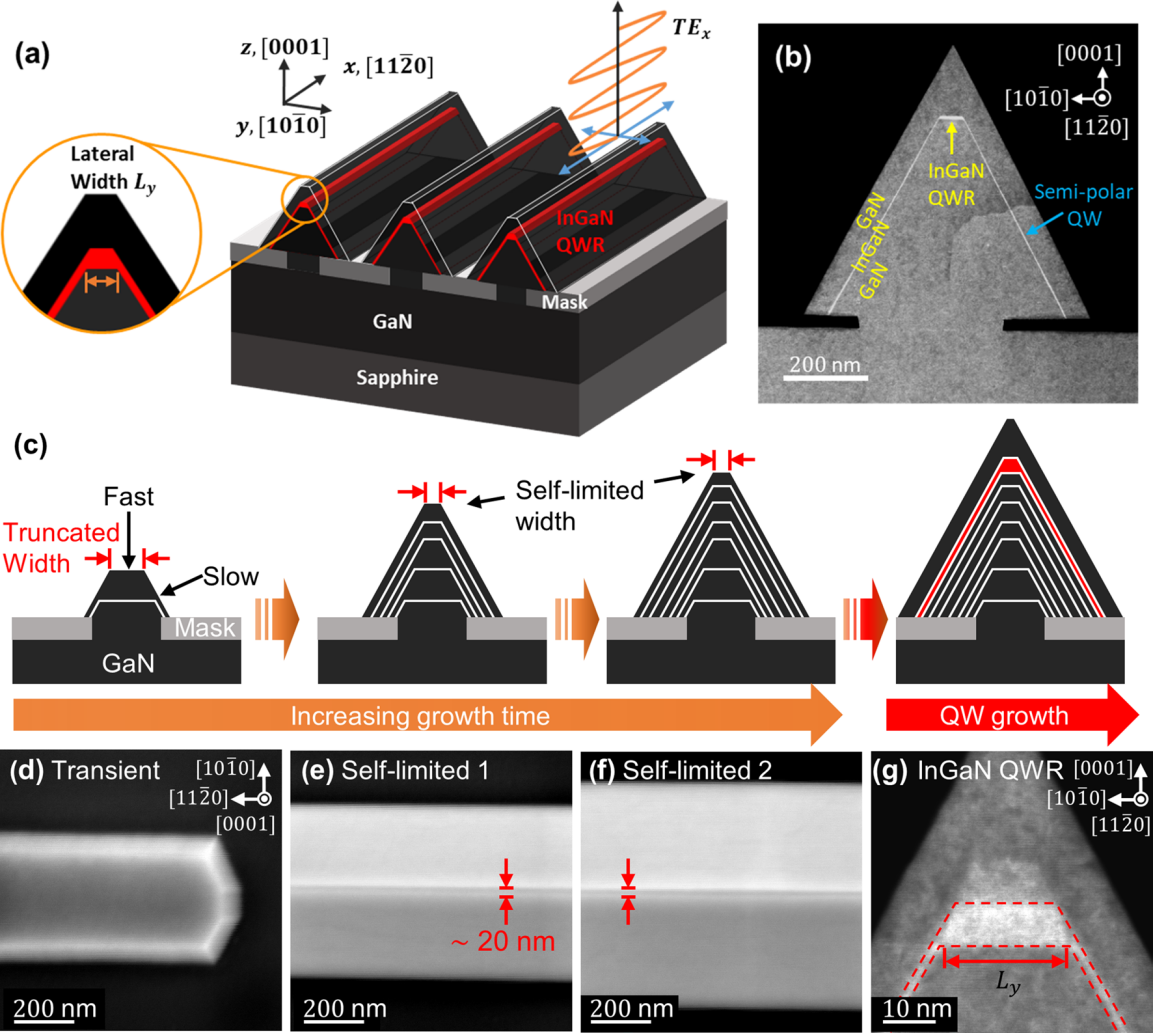
(**a**) 3D schematic of one-dimensional InGaN QWR array formed on apex of GaN sub-micron prism structures along $\left[ {11\overline{2}0} \right]$ direction. Orange circle: a cross-sectional view of single InGaN QWR with finite lateral width. (**b**) Cross-sectional TEM image of the structure. (**c**) Schematic of morphological evolution of GaN core under self-limited growth regime and regrowth of single InGaN and GaN capping layers. Red arrows: truncation width on *c*-plane facet. White lines: morphology of structure at different growth times. Top-view SEM images of structural morphologies at stages of (**d**) early growth (transient), (**e**) complete prism shape, and (**f**) self-limited growth regime. (**g**) Cross-sectional TEM image of single InGaN layer grown on self-limited structure.

Figure 1a shows a 3D sketch of an InGaN QWR formed on a GaN triangular prism structure. We defined the x, y, and z coordinates as $\left[ {11\overline{2}0} \right]$, $\left[ {10\overline{1}0} \right]$, and $\left[ {0001} \right]$, respectively. A vertical (lateral) quantum confinement exists along the z (y) direction. SEM images of the array of the GaN prism structures are shown in Supporting Information S1. Cross-sectional transmission electron microscope (TEM) images show that an InGaN layer successfully formed on the prism structure (Fig. 1b). We defined the InGaN layer located at the apex of the structure as a QWR and the lateral width of the QWR as the bottom length of the trapezoidal shape.

Figure 1c shows our control of the lateral width of a single QWR on a 3D structure by using a self-limited growth mechanism, by which the evolution of the surface profile is limited when reaching a thermodynamically steady-state, allowing us to control the morphology and size of the quantum structure^22–24^. In convex growth mode, a faster growth rate at the apex than that at the nearby crystal facet is essential to achieving a finite width under this growth mechanism^24^. The structure preserves the finite truncation despite the increase in growth time after the growth condition reaches the self-limited growth regime. This finite truncation at the apex determines the lateral width of the InGaN QWR.

Figure 1d–f shows top-view scanning electron microscope (SEM) images of the structure with an increase in growth time. At the early stage growth, both *c*-plane (0001) and semi-polar planes $\left\{ {10\overline{1}1} \right\}$ are simultaneously formed (Fig. 1d). Our growth conditions (see Sample Information) exhibit faster growth rates on the *c*-plane than that on the semi-polar planes. We observed a finite truncation at approximately a 20 nm scale at the apex of GaN sub-micron prism structures. Notably, its width does not change as the growth time proceeds (Fig. 1e–f) and we controlled the width with varying growth temperature (see Supporting Information S2). These results imply that our core growth condition is within the self-limited growth regime. Figure 1g also shows that the width is the same after growing the InGaN layer. Owing to the growth rate anisotropy between the *c*-plane and the semi-polar planes, we achieved a much thicker InGaN layer on the *c*-plane (~ 7 nm) compared to the thicknesses of the semi-polar planes (< 1 nm).

### Position-dependent CL measurements for InGaN layer on self-limited structure

**Figure 2:**
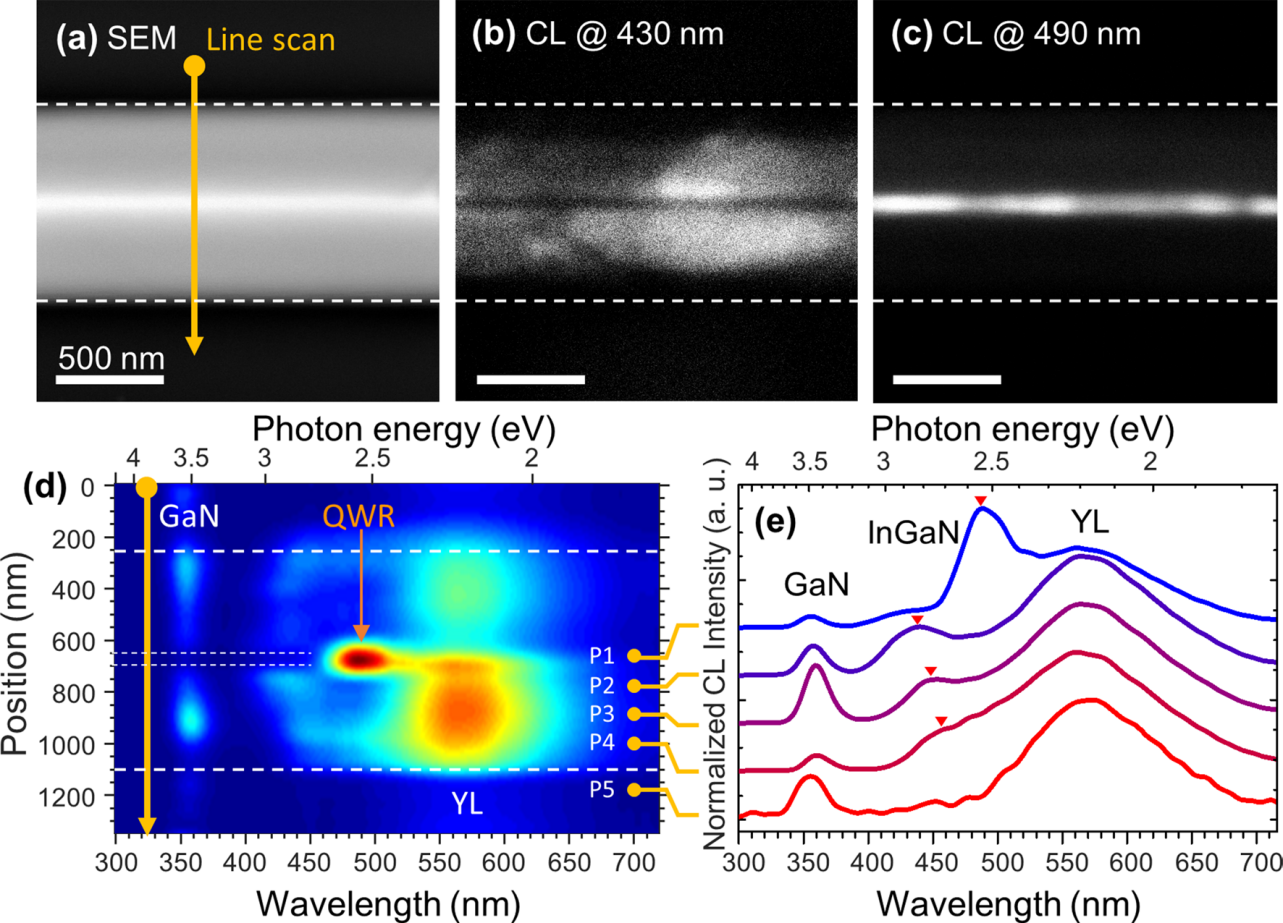
Position-dependent luminescence of single InGaN layer on self-limited prism structure. (**a**) Top-view SEM image. Yellow arrows: line scanning positions for acquiring CL spectra. Monochromatic CL images acquired at (**b**) 430 and (**c**) 490 nm. Bold white-dashed lines: bottom edge positions of structure in corresponding SEM image. (**d**) Line-scanned CL spectra across the yellow arrow in (**a**). Thin dotted lines: boundary of QWR. (**e**) CL spectrum collected from apex (P1), semi-polar facet (P2–4), and outside structure (P5). Red triangles: peak emission wavelengths of InGaN layer at each point.

We conducted cathodoluminescence (CL) measurements to examine the position-dependent optical characteristics of the structure. Figure 2a–c shows a SEM image and monochromatic CL images at center wavelengths of 430 and 490 nm, respectively. The QWs on the semi-polar planes have shorter emission wavelengths and a relatively lower intensity at the center of the structure. The center exhibits a strong emission in the 490 nm CL image (Fig. 2c), which is exactly matched with the central dark line in the 430 nm CL image (Fig. 2b). Monochromatic CL images clearly distinguish the emission position depending on the wavelength.

Figure 2d,e shows the position-dependent (line-scanned) CL spectra across the structure and provides more detailed optical properties. As a reference point, the CL spectrum measured outside of the structure (P5) shows only two peaks at approximately 360 and 570 nm, corresponding to the band edge emission and defect-related yellow luminescence (YL) of GaN^25^, respectively. For other positions (P1–P4), an InGaN-layer related emission arises in comparison to the reference spectrum (P5). The emission wavelengths of the semi-polar planes show a slight blueshift by changing the position from bottom (P4) to top (P2) (see Supporting Information S3). At the center of the structure (P1), we observed that the emission wavelength is dramatically red-shifted by ~ 50 nm for P2. We obtained a huge emission energy difference of ~ 250 meV between them, indicating that a single InGaN QWR is energetically well-isolated from the InGaN QW on the semi-polar planes with sufficient potential barriers.

A QW thickness variation and quantum-confined Stark effect depending on the crystal orientation may explain the position-dependent emission characteristics. As seen in Fig. 1g, the QW on a semi-polar facet (~ 1 nm) is much thinner than that on a polar facet (~ 7 nm). A thinner QW experiences a greater quantum confinement, inducing a higher energy state. Moreover, the piezoelectric built-in electric field along the *c*-plane is also higher than that along the semi-polar planes^26^. Considering these two aspects, the significant decrease in emission energy in the QWR can be explained.

### Polarization-resolved μ-PL with various lateral dimensions of InGaN quantum structures

We carried out polarization-resolved μ-PL measurements for a single InGaN QWR to investigate the polarization anisotropy with the degree of linear polarization (DLP), which is defined as follows:

$${\text{DLP}} = \frac{{{\text{I}}_{max} - {\text{I}}_{min} }}{{{\text{I}}_{max} + {\text{I}}_{min} }},$$

where ${\text{I}}_{max} ({\text{I}}_{min} )$ is the maximum (minimum) integrated PL intensity. We compared the DLP of three samples: two InGaN QWR samples grown on a self-limited prism structure with lateral widths of approximately 22 and 55 nm, respectively, and an InGaN QW sample on *c*-plane with a large truncated (> 200 nm) hexagonal pyramid structure as a reference. We designed the reference to show the conventional features of a QW on *c*-plane, which does not exhibit an in-plane polarization anisotropy, in contrast to the case of QWs on the *a*-plane and *m*-plane^5^.

**Figure 3:**
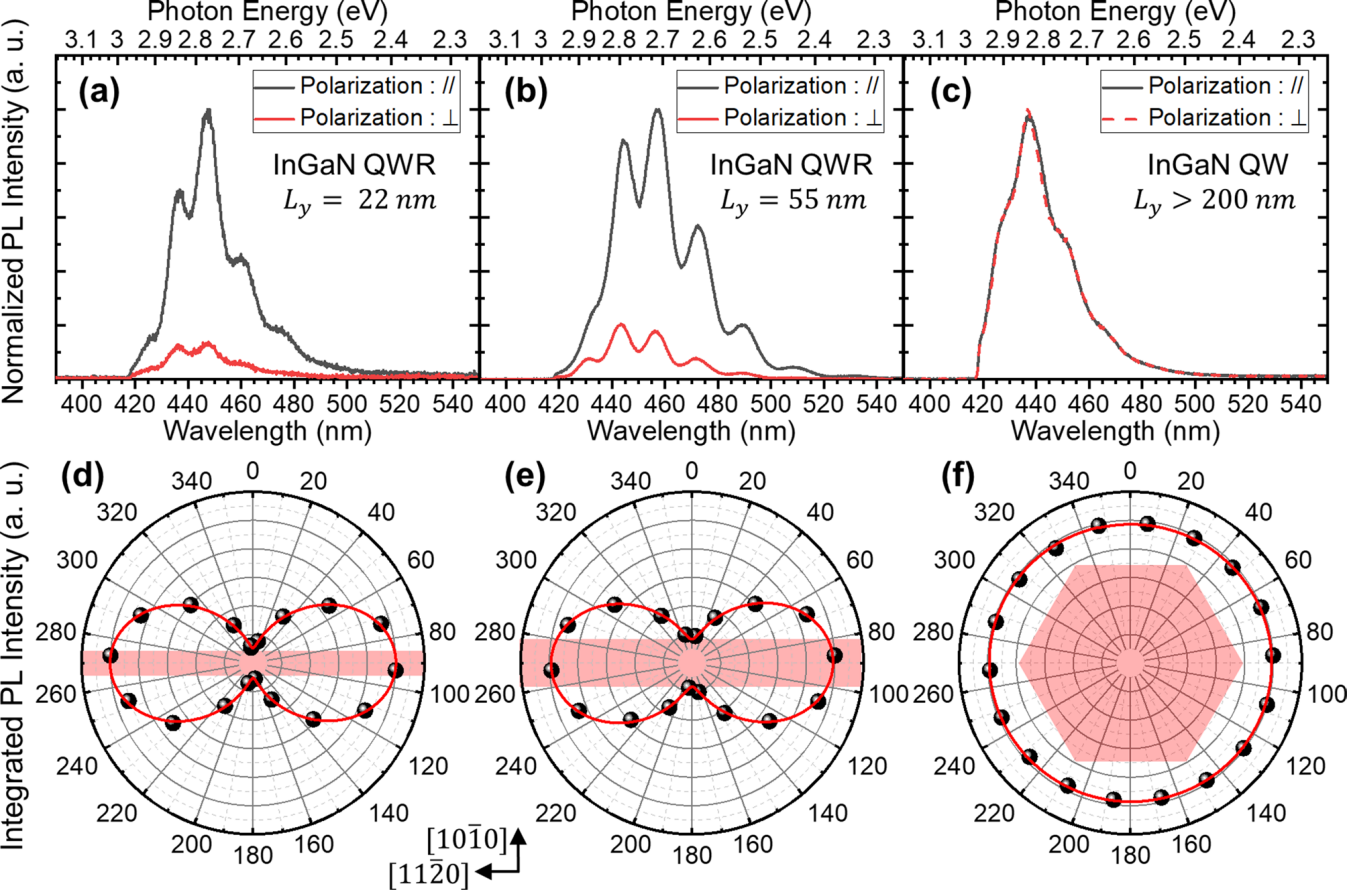
Polarization-resolved μ-PL data at room temperature with various InGaN quantum structures. $L_{y}$: lateral width of QWR as specified in schematic of Fig. 1a. μ-PL spectra of InGaN QWRs with lateral width of (**a**) 22 and (**b**) 55 nm, and (**c**) InGaN QW grown on large truncated (> 200 nm) hexagonal pyramid. Gray (red) lines: PL spectra depicted with polarization direction parallel (perpendicular) to InGaN QWR direction $\left[ {11\overline{2}0} \right]$. (**d**–**f**) Integrated PL intensity from each spectrum with various detection angles from (**a**–**c**). Experimental data (black dots) are fitted with sine square function (solid red lines). Shaded areas: conceptual images of investigated quantum structures.

Figure 3a,b shows the polarization-resolved μ-PL spectra of QWRs with lateral widths of ~ 22 and ~ 55 nm, measured along both the parallel and perpendicular directions of the QWR at room temperature. A much higher intensity along the parallel direction was observed as compared to the perpendicular direction for both QWRs. In contrast, no polarization dependence was observed for the reference QW sample, as expected (Fig. 3c). We plotted the integrated PL intensity with various polarization detection angles, as shown in Fig. 3d,f. The InGaN QWR samples with 22 and 55 nm width exhibit a strong polarization anisotropy of approximately 80% and 71%, respectively. Two reasons are possible to explain the polarization anisotropy: a 1D QWR geometry-induced energy structure modification, and the photonic structural effect due to the 1D GaN prism structure. If the photonic structural effect is dominant, a structural volume dependence on the DLP will occur. For instance, semiconductor nanowires exhibit a significantly large optical anisotropy^27^, their size dependency of which has previously been reported^28^. We measured the DLP using two InGaN QWRs grown on two different base sizes of the GaN core prism structures but with the same finite truncation (see Supporting Information S4). However, we were unable to observe a notable difference between them, indicating that the photonic structural size effect is not dominant for the high DLP observed in our system. Therefore, in the following we analyze and discuss the optical properties in terms of the electronic energy structures of the QWR.

### Calculation of energy eigenstates and optical transition matrix elements based on k∙p perturbation theory

We elucidate the high polarization anisotropy of an InGaN QWR from two perspectives: (i) anisotropic strain and (ii) asymmetric quantum confinement applied to the QWR. To clarify these features from two perspectives, we calculated the energy eigenstates and inter-band optical transition matrix elements using a 6-band k·p perturbation theory (see details in the Calculation Information section). Next, we suggest a way to investigate the energy structures of an InGaN QWR based on the concept of an effective energy spacing analogous to the *m*-plane QW.

**Figure 4:**
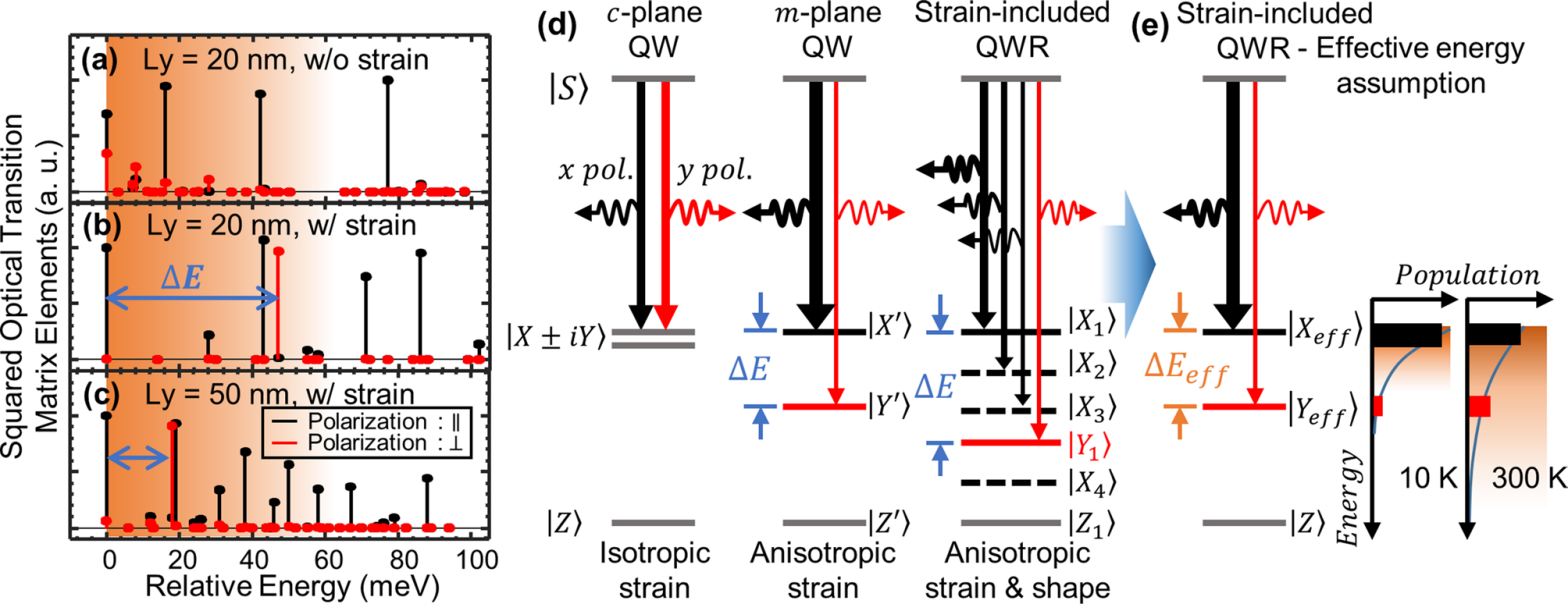
(**a-c**) Squared optical transition matrix elements as function of relative energy difference from ground state depending on effects of strain and lateral width of QWR. *L*_*y*_ = 20 nm QWR (**a**) without and (**b**) with strain, and (**c**) *L*_*y*_ = 50 nm QWR with strain. Gradually shaded area: rough indication of extent of thermally activated energy states at 300 K following Boltzmann distribution. (**d**) Schematics of energy configurations of QW for various crystal directions and strain-included QWR. Strain-included QWR possesses more sublevels owing to additional quantum confinement. X (Y): direction parallel (perpendicular) to QWR. $\Delta {\text{E}}$: energy level spacing between two-hole states having orthogonal polarization bases. (**e**) Effective energy assumption for strain-included QWR, analogous to anisotropic strained *m*-plane QW.

Figure 4a,b shows how the existence of the strain changes the optical transition matrix elements of the QWR as a function of energy relative to the ground state transition. In the absence of strain (Fig. 4a), only an asymmetric quantum confinement from the 1D geometry induces a valence band mixing, which causes a non-identical portion of the parallel and perpendicular polarization of the QWR at a ground state. However, the ratio is insignificant compared to a strain-included QWR, in which most of the transition states are strongly polarized as shown in Fig. 4b. The first perpendicular polarization-dominant transition arises at ~ 47 meV higher than the ground state, whereas others mostly have a parallel polarization with a smaller energy spacing.

In general, the energy spacing of the confined states depends on the width of a confinement potential. For a wider QWR (Fig. 4c), the spacing decreases, and the first perpendicular component appears at 18 meV higher. As an interesting feature, the overall configurations of the matrix elements for a strain-included 20 nm QWR (Fig. 4b) and a 50 nm QWR (Fig. 4c) are quite similar. This indicates that the effect of the strain significantly changes the valence band structure. In addition, the lateral dimension of the QWR mainly varies the energy spacing among the states. Therefore, we deduced that the strain anisotropy caused by the 1D geometry of the QWR, rather than an asymmetric quantum confinement effect, plays a dominant role in the high polarization anisotropy.

Figure 4d shows the differences in energy configurations of the strain-included QWR and the QWs on the two different types of crystal plane. Compared to the unpolarized *c*-plane QW, the *m*-plane QW has an anisotropic strain, which gives rise to the strongly polarized hole states with a finite energy level spacing^5^. Strain-included QWR exhibits similar features to the *m*-plane QW. A difference is that the QWR possesses more sublevels owing to the additional quantum confinement (i.e., vertical and lateral directions). Because the contributions of the higher energy states to the optical transition are weak by the thermal distribution, only a few sublevels are involved. Therefore, we presumed that the energy structure of a strain-included QWR contains two representative effective hole states having an orthogonal polarization, as represented in Fig. 4e. The temperature dependence on the DLP of the strain-included QWR would be analogous to that of the *m*-plane QW.

It is possible to measure the polarization anisotropy directly of each energy state through a photoluminescence excitation experiment^11^. However, because the material inhomogeneity of InGaN is inevitable, resolving each higher energy state is quite a challenge^29^. Measuring the temperature-dependent degree of polarization by integrating the PL spectrum is one of the ways to investigate the energy level spacing between orthogonal polarization bases^30^.

### Temperature dependence of InGaN QWR with various lateral widths

**Figure 5:**
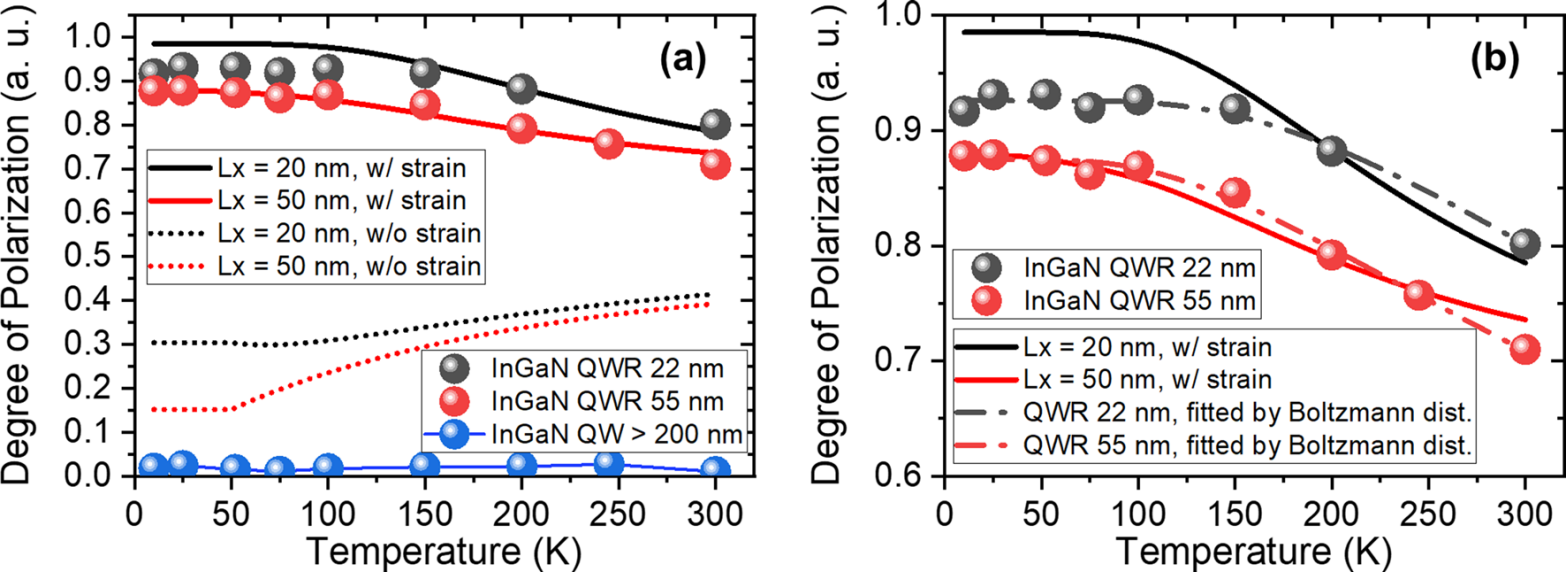
Measured and calculated temperature-dependent degree of polarization for two different lateral dimensions. (**a**) Calculated degree of polarization by summing all matrix elements at different temperatures in presence (solid lines) and absence (dotted lines) of strain. Such degrees of polarization are corrected through a Boltzmann distribution. Filled circles: measured data for InGaN QWRs (22 and 55 nm) and InGaN QW on large truncated pyramid. (**b**) Enlarged graph of (**a**) with experimental data directly fitted through Boltzmann distribution (dash-dotted lines).

To investigate the effect of the width and the strain of the QWRs on their energy level spacing, we measured the DLP as a function of temperature with various lateral sizes of the InGaN quantum structure, as shown in Fig. 5a. To measure the effective energy level spacing (${\Delta E}_{{{\text{eff}}}}$) between both orthogonally polarized states, we fitted the temperature-dependent DLP with the Boltzmann statistics^30^ as follows:

$${\text{P}} = {\text{P}}_{0} \left[ {1 - Aexp\left( {\frac{{{\Delta }E_{eff} }}{{k_{B} T}}} \right)} \right],$$

where ${\text{P}}_{0}$ is the initial DLP at low temperature, A is a fitting parameter, and ${\text{k}}_{{\text{B}}} {\text{T}}$ is the thermal energy at a certain temperature. For simplicity, we consider that an inhomogeneous InGaN alloy only contributes to a broadening of each energy state and does not create new eigenstates (i.e., quantum dot-like discrete energy states). Therefore, the summation of all matrix elements represents an equation of the entire PL spectrum.

Figure 5a shows the temperature dependence of the calculated DLP using matrix elements for two different widths of the QWR, in the presence (solid lines) and absence (dotted lines) of strain, together with that of the measured DLP from integrated PL intensities (symbols). In the absence of strain, the QWRs exhibit a 30% (15%) DLP for a 20 nm (50 nm) wide QWR at low temperatures. These values are similar to that of a GaAs/AlAs QWR formed in the V-grooves, which was solely described through an asymmetric quantum confinement owing to a negligible lattice mismatch^12^. The values gradually increase with varying temperature from 10 to 300 K. However, in the presence of strain, its temperature dependence is opposite to the previous case. The 20 nm wide QWR shows a strong polarization anisotropy (98%) at low temperature, which is higher than that of the 50 nm wide QWR (87%). The DLPs gradually decrease with an increase in temperature for both widths of QWRs. This is reasonable because the perpendicularly polarized state at a higher energy is thermally activated with increasing temperature. We clearly observed that the tendency of the experimental data coincides with the calculation when taking the presence of the strain into account.

Figure 5b shows both the experimental data (symbols) and the fitted lines with a Boltzmann distribution (dash-dotted lines). We found that the 22 nm wide QWR has a strong and robust (i.e., less temperature-dependent) polarization anisotropy than the 55 nm wide QWR over the entire temperature range. We obtained ${\Delta E}_{{{\text{eff}}}}$ by directly fitting the experimental data as 58.7 ± 8.5 and 41.3 ± 5.0 meV for the 22 and 55 nm wide QWRs, respectively. In comparison, we also derived ${\Delta E}_{{{\text{eff}}}}$ from the calculated DLP, which are solid lines in Fig. 5a,b, as 36.6 ± 0.6 and 25.5 ± 0.2 meV for the 20 and 50 nm wide QWRs, respectively. The results show the differences between the values from the experiment and calculation. We speculated the reason for this as the designed geometry and the size for the calculation (i.e., square-shaped InGaN QWR hosted by the sufficiently large GaN cube) somehow differing from the real geometry (i.e., trapezoidal-shaped InGaN QWR hosted by the GaN prism). Thus, the shape of the lateral confining potential and strain distribution near the QWR might provide the difference. According to the experimental and calculated data, the narrow width of the QWR shows a larger effective energy level spacing than the large width. It is worth noting that the effective energy level spacing ${\Delta E}_{{{\text{eff}}}}$ is attributed to the quantum confinement by the lateral width of the QWR. Therefore, we confirmed that the lateral quantum confinement from the wire geometry determines the robustness of the degree of polarization with an increase in temperature.

In conclusion, we demonstrated a strong and robust optical polarization anisotropy of a site-selective and size-controlled III-nitride single QWR, reaching an 80% degree of polarization at room temperature. To investigate the anisotropic properties stemming from a 1D geometry, we grew the QWR on the *c*-plane of a GaN prism structure to exclude any inherent strain anisotropy from the crystal direction. Owing to our ability to control the lateral width of the QWR using a self-limited growth mechanism, we investigated this size-dependent optical polarization anisotropy from two perspectives, namely, an asymmetric quantum confinement effect and an anisotropic strain distribution from the 1D geometry. According to the 6-band k·p theory calculation, the polarization anisotropy is significantly associated with the presence of the strain, which strongly polarizes the hole states with a finite energy level spacing. We adapted the concept of effective energy level spacing to describe the robustness of the degree of polarization in the QWR emission against temperature. We showed that a smaller width of the QWR (i.e., a more 1D QWR) exhibits better performances than a larger width. We expect that improving the degree of polarization and its robustness is viable through fabricating a narrower width of QWR under the mechanism. Therefore, we believe that our light-emitting platform, based on III-nitride QWRs, is a promising candidate for future photonic applications relying on the intensive use of the optical polarization, including polarizer-free display, polarization-sensitive sensing and imaging.

## Methods

### Sample information

The array of triangular GaN prism and a single InGaN QWR were grown on the *c*-axis GaN/sapphire substrate using showerhead-type 19 × 2′ metal–organic chemical vapor deposition (MOCVD). Stripe patterned 30 nm-thick SiO_2_ mask aligned to $\left[ {11\overline{2}0} \right]$ direction was prepared by electron beam lithography for the selective area growth. An opening width was 300 nm, and a pitch was 2 μm to investigate a single structure through μ-PL experiments. A 22 and 55 nm truncation widths at the apex of the structure were achieved under the growth temperature of 950 °C and 980 °C, respectively. InGaN and GaN capping layer were grown under a nitrogen atmosphere at 720 °C and 850 °C, respectively. We use trimethylgallium (TMGa) for the self-limited structure and triethylgallium (TEGa) and trimethylindium (TMI) for the InGaN and the GaN capping layer. In order to clearly show the position-dependent emission characteristics through CL measurements, we prepared another sample exhibiting strong CL intensity (see Supporting Information S5).

### Structural and optical characterization

The monochromatic CL images and position-dependent emission spectra were observed by the CL system (Gatan, Mono4) installed in SEM (FEI XL30). μ-PL setup with an objective lens (× 50, N.A. 0.65) was used for polarization-resolved PL measurement using the linear polarizer and the half-wave plate. The sample was mounted in a cryostat (MONTANA instruments) for temperature-dependent μ-PL from 10 to 300 K. The excitation laser sources were 405 nm laser diode to excite InGaN QWR. The cross-sectional TEM image and energy dispersive X-ray spectroscopy (EDS) data were measured by spherical aberration-corrected scanning TEM (JEOL-ARM200F, 200 kV).

### Calculation information

We designed that a square-shaped InGaN QWR along the $\left[ {11\overline{2}0} \right]$ direction (x-direction) was embedded in the homogeneous GaN cube with periodic boundary conditions. We set 20 and 50 nm-wide InGaN QWRs with the 7 nm thickness containing 15% of indium composition obtained by EDS. We use the 6-band Hamiltonian written in $\left| {\text{X}} \rangle \right., \left| {\text{Y}}\rangle \right.$ and $\left| {\text{Z}} \rangle \right.$ bases including spin–orbit coupling described by Chuang et al*.*^31^ with the material parameters of Park et al*.*^32^ The DLP is defined by summation of all the square of transition matrix:

$${\text{DLP}} = \frac{{\sum \left| {M_{\parallel }^{ij} } \right|^{2} - \sum \left| {M_{ \bot }^{ij} } \right|^{2} }}{{\sum \left| {M_{\parallel }^{ij} } \right|^{2} + \sum \left| {M_{ \bot }^{ij} } \right|^{2} }},$$

where $M_{\parallel }^{ij} \,\left( {M_{ \bot }^{ij} } \right)$ is the transition matrix element from i-th electron state the j-th hole state along parallel (perpendicular) to QWR, which is given with dipole approximation by

$$M_{\parallel , \bot }^{ij} = \langle \psi_{e}^{j} {\text{|p}}_{\parallel , \bot } {|}\psi_{h}^{i}\rangle ,$$

with electron $\left| {\psi_{e}^{j} } \rangle\right.{ }$ and hole $\left| {\psi_{h}^{i} }\rangle \right.{ }$ wavefunction, and the momentum operator **p**.

## Supplementary information

Supplementary file1
