# Supplementary material for: Strong and robust polarization anisotropy of site- and size-controlled single InGaN/GaN quantum wires
Source: Sci Rep. 2020 Sep 21;10:15371. doi: 10.1038/s41598-020-71590-x (PMC7505962; doi:10.1038/s41598-020-71590-x)
Supplement: Supplementary file 1 — Supplementary file1 [file 41598_2020_71590_MOESM1_ESM.docx]

Supporting Information

Strong and robust polarization anisotropy of site- and size-controlled single InGaN/GaN quantum wires

*Hwan-Seop Yeo^1^, Kwanjae Lee^1^, Young Chul Sim^1^, Seoung-Hwan Park^2^, and Yong-Hoon Cho^1*^*

*^1^ Department of Physics and KI for the NanoCentury, Korea Advanced Institute of Science and Technology (KAIST), Daejeon 34141, Republic of Korea*

*^2^Department of Electronics Engineering, Catholic University of Daegu, Kyeongsan 38430, Republic of Korea*

^*^corresponding author email: yhc@kaist.ac.kr

**S1. Array of triangular GaN prism structures under self-limited growth mechanism**


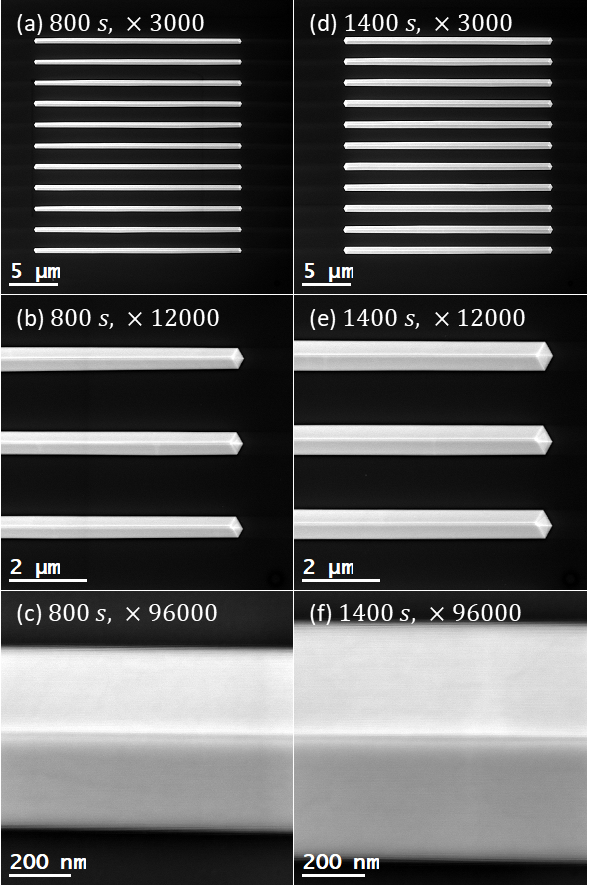


Supporting Figure S1. SEM images of array of triangular GaN prism structure under self-limited growth mechanism with two different growth time at (a-c) 800 s and (d-e) 1400 s.

**Supporting Notes 1|** The array size of 300 nm-wide striped pattern is approximately $20\times20 \mu m$, the direction of which is aligned to $[11\overline{2}0]$. The bottom widths of triangular GaN prism structures are quite uniform within the array. Despite different growth time (800 and 1400 s), Figure S1c and S1f show the same finite truncation. All the structures possess the finite truncation at the apex, as shown in Figure S1b and S1e. These SEM images support that our growth condition is under the self-limited growth regime.

**S2. Control of finite truncation at apex of GaN prism structure with various growth temperature**


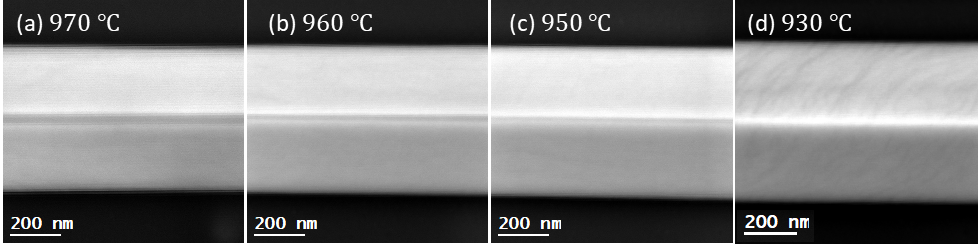


Supporting Figure S2. SEM images of finite width at apex of GaN prism structure with different growth temperatures. (a) 970 $\mathbf{℃}$, (a) 960 $\mathbf{℃}$, (a) 950 $\mathbf{℃}$, and (a) 930 $\mathbf{℃}$.

**Supporting Notes 2**| We confirmed that our growth condition is under the self-limited by controlling the finite width at the apex of the structure with varying growth temperature. The growth rate for each crystal plane is related to the surface energy and the stability of surface atom. The stable crystal plane exhibits a slower growth rate than an unstable plane in general. In the case of GaN, Ga (N) terminated crystal plane becomes stable at high (low) growth temperatures. Since the c-plane and the semi-polar plane are Ga and N-nitrogen terminated, respectively, the c-plane (semi-polar plane) becomes stable at the high (low) growth temperature. Therefore, the relative growth rates of the semi-polar plane and c-plane are changed in opposite directions as varying growth temperature. For instance, the growth rate of the c-plane and the semi-polar plane becomes slower and faster at the high growth temperature.^1^ According to the mechanism, the group-III adatom diffusion kinetics and growth rate anisotropy between two different crystal directions determines the finite width. Therefore, a growth temperature is one of the major parameters which may vary the width at the apex. Figure S2a-c show the width of finite truncations are ~46, ~26, and ~20 nm for the structures grown at 970, 960, and 950 $℃$, respectively. We were unable to observe the finite truncation at the structure grown at 930 $\mathbf{℃}$, as shown in Figure S2d. As increasing growth temperature, we observed that the finite widths become wider and exist over the array of the prism structure. These results show that the possibility of control of the finite width with a few tens of nanometer scale, and our growth condition is under the self-limited growth regime.

**S3. Structural analysis of the InGaN quantum wire (QWR) on the self-limited structure**


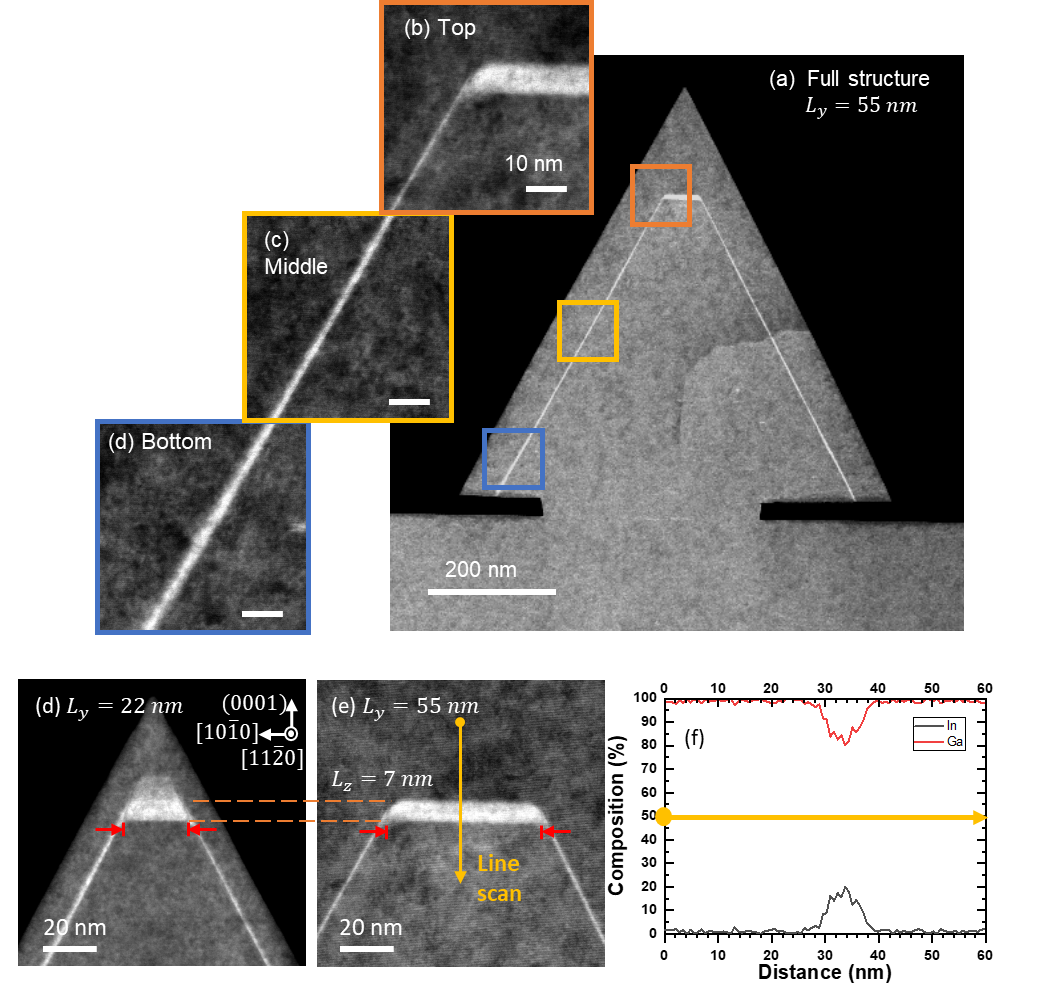


Supporting Figure S3. (a) TEM image of the InGaN QWR embedded in GaN prism structure. TEM images of InGaN layer at (b) Top (near quantum wire), (c) middle and (d) bottom of the structure. Enlarged TEM images of (d) 22 and (e) 55 nm width of InGaN QWR.
(f) Composition of the structure obtained through energy-dispersive X-ray spectroscopy.

**Supporting Notes 3**| We measured TEM images of the InGaN layer embedded in GaN prism structure at various positions. Figure 3Sa shows a full image of the structure. We observed that the InGaN layer is successfully formed on the self-limited structure. From bottom to top, the thickness of an InGaN layer on a semi-polar plane becomes thinner, as shown in Figure S3b-d. In general, a thinner quantum well exhibits a stronger quantum confinement effect, which gives rise to a blueshift of emission peak wavelength. This feature may explain the blueshift of CL emission of InGaN QW on the semi-polar plane from bottom to top of the structure, as shown in position-dependent CL (Figure 2). We confirmed that the thickness of the InGaN QWRs is ~7 nm. We obtained the indium composition as ~15 % through energy dispersive X-ray spectroscopy measurement.

**S4. Polarization-resolved** $\boldsymbol{\mu}$**-PL depending on size of GaN prism structure.**


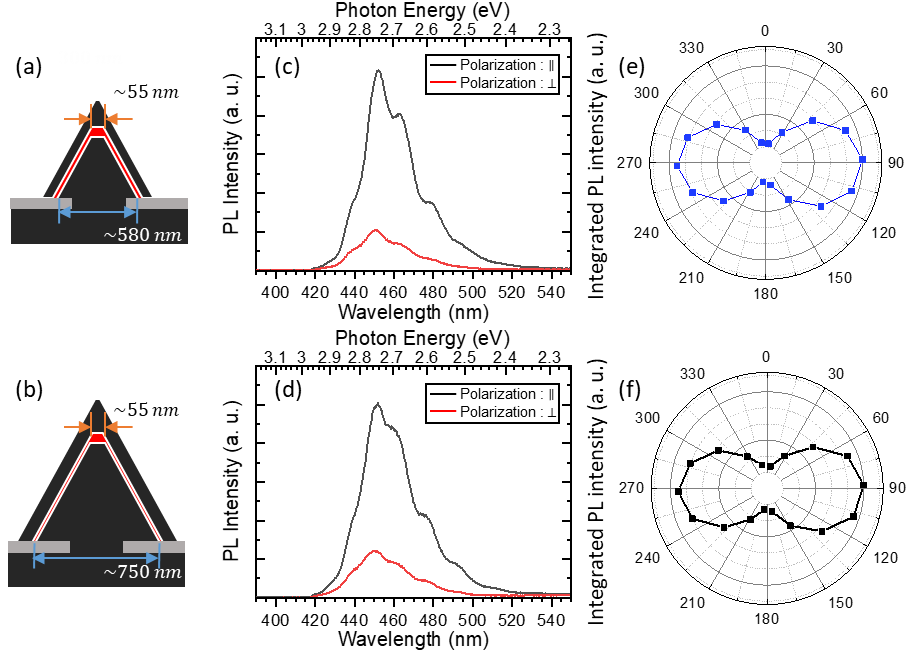


**Supporting Figure S4.** Schematic of two different sizes of GaN prism structure with same width of InGaN QWR. (a) ~580 and (b) ~750 nm. (c-d) Polarization-resolved $\mu$-PL spectra of QWR at room temperature of (a-b). (e-f) Integrated PL intensity of (a-b) with various detection angles.

**Supporting Notes 4**| We measured a degree of linear polarization (DLP) for two different sizes of GaN prism structure to investigate the photonic effect of the structure. Figure S5c and S5d show a similar intensity ratio of the parallel to perpendicular polarization. We extract DLP by fitting a sine square function for both cases. We obtain 67.5% (64%) of DLP for the GaN prism size of ~580 nm (~750 nm). The results show that the size of GaN prim provides an insignificant photonic structural effect on DLP of QWR emission.

**S5. Cathodoluminescence (CL) measurement for two different samples**


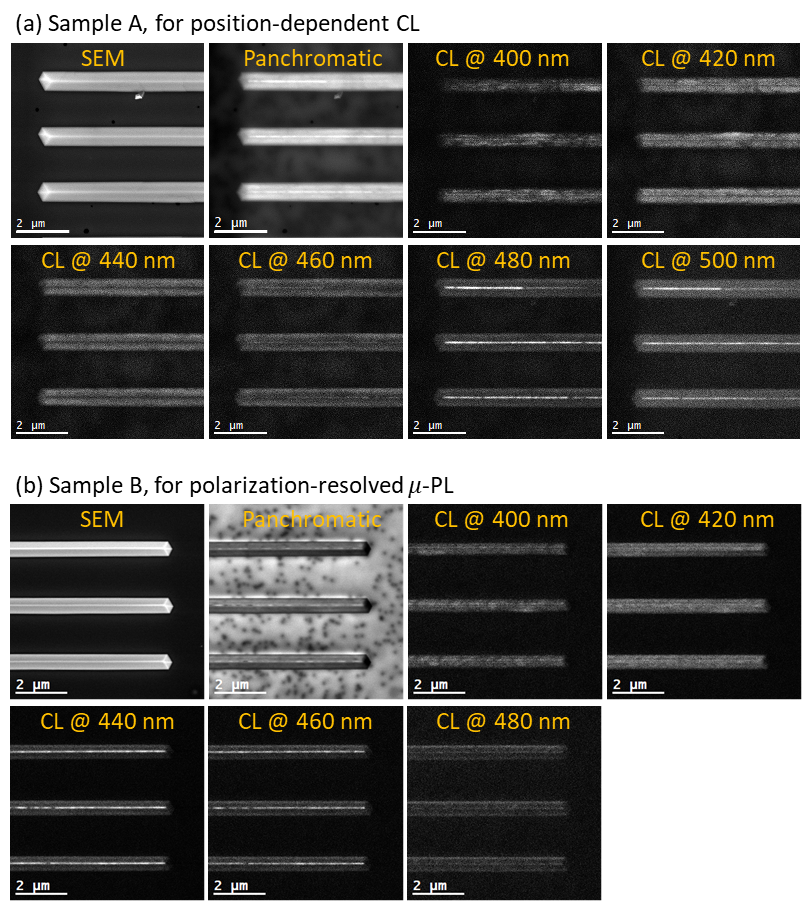


Supporting Figure S5. SEM, panchromatic and monochromatic images of two different samples for (a) position-dependent CL (Figure 2) and (b) polarization-resolved $\boldsymbol{\mu}$-PL measurement (Figure 3 and 5).

Reference

1 Hiramatsu, K. *et. al.* Recent Progress in Selective Area Growth and Epitaxial Lateral Overgrowth of III-Nitrides Effects of Reactor Pressure in MOVPE Growth. *Phys. stat. sol. (a)* **176**, 535 (1999).
